# Novel Loss-of-Function Mutations in *NPR2* Cause Acromesomelic Dysplasia, Maroteaux Type

**DOI:** 10.3389/fgene.2022.823861

**Published:** 2022-03-16

**Authors:** Jing Wu, Mengru Wang, Zhouyang Jiao, Binghua Dou, Bo Li, Jianjiang Zhang, Haohao Zhang, Yue Sun, Xin Tu, Xiangdong Kong, Ying Bai

**Affiliations:** ^1^ Department of Pediatrics, First Affiliated Hospital of Zhengzhou University, Zhengzhou, China; ^2^ Key Laboratory of Molecular Biophysics of the Ministry of Education, College of Life Science and Technology and Center for Human Genome Research, Huazhong University of Science and Technology, Wuhan, China; ^3^ Department of Vascular and Endovascular Surgery, First Affiliated Hospital of Zhengzhou University, Zhengzhou, China; ^4^ Department of Physiology and Neurobiology, School of Basic Medical Sciences, Zhengzhou University, Zhengzhou, China; ^5^ Department of Endocrinology, First Affiliated Hospital of Zhengzhou University, Zhengzhou, China; ^6^ Genetic and Prenatal Diagnosis Center, Department of Obstetrics and Gynecology, First Affiliated Hospital of Zhengzhou University, Zhengzhou, China

**Keywords:** acromesomelic dysplasia, maroteaux type, natriuretic peptide receptor 2, loss-of-function mutation, growth hormone therapy, genotype analysis

## Abstract

Acromesomelic dysplasia, Maroteaux type (AMDM) is a rare skeletal dysplasia characterized by severe disproportionate short stature, short hands and feet, normal intelligence, and facial dysmorphism. Homozygous or compound heterozygous mutations in the natriuretic peptide receptor 2 (*NPR2*) gene produce growth-restricted phenotypes. The current study was designed to identify and characterize *NPR2* loss-of-function mutations in patients with AMDM and to explore therapeutic responses to recombinant growth hormone (rhGH). *NPR2* was sequenced in two Chinese patients with AMDM via next generation sequencing, and in silico structural analysis or transcript analysis of two novel variants was performed to examine putative protein changes. rhGH treatment was started for patient 1. Three *NPR2* mutations were identified in two unrelated cases: two compound heterozygous mutations c.1112G>A p.(Arg371Gln) and c.2887+2T>C in patient 1 and a homozygous mutation c.329G>A p.(Arg110His) in patient 2, yielding distinct phenotypes. RNA extracted from peripheral blood cells of patient 1 showed alternatively spliced transcripts not present in control cells. Homology modeling analyses suggested that the c.1112G>A p.(Arg371Gln) mutation disrupted the binding of NPR-B homodimer to its ligand (C-type natriuretic peptide) in the extracellular domain as a result of global allosteric effects on homodimer formation. Thus, c.2887+2T>C and c.1112G>A p.(Arg371Gln) in *NPR2* were loss-of-function mutations. Furthermore, rhGH therapy in patient 1 increased the patient’s height by 0.6SDS over 15 months without adversely affecting the trunk-leg proportion. The short-term growth-promoting effect was equivalent to that reported for idiopathic short stature. Overall, our findings broadened the genotypic spectrum of *NPR2* mutations in individuals with AMDM and provided insights into the efficacy of rhGH in these patients.

## 1 Introduction

Acromesomelic dysplasia is a heterogeneous group of rare chondrocyte dysfunctions affecting the distal and middle segments of the extremities. Acromesomelic dysplasia occurs in isolated abnormal bone growth and skeletal morphology and is associated with genital and neurological disorders (Mustafa et al., 2020; Khan et al., 2016). To date, five types of acromesomelic dysplasia, i.e., acromesomelic dysplasia, Maroteaux type (AMDM, OMIM #602875); Grebe dysplasia (OMIM #200700) (Umair et al., 2017; Ullah et al., 2018); fibular hypoplasia andcomplex brachydactyly type (Du pan, OMIM #228900)) (Mortier et al., 2019); acromesomelic dysplasia Osebold-Remondini type (OMIM #112910) (Osebold et al., 1985; Ullah et al., 2018); and another recently discovered novel type (OMIM #609441) (Díaz-González et al., 2022), have been identified. These diseases are distinct entities because of their unique features and have been shown to be caused by four genes, namely, natriuretic peptide receptor 2 (*NPR2*), growth and differentiation factor-5, bone morphogenetic protein receptor-1b, protein kinase cGMP-dependent type II, showing autosomal recessive inheritance. Among disease types, the Osebold-Remondini type has not yet been genetically mapped. However, diagnosis is generally made using clinical, radiological, and genetic information.

Specific mutations in *NPR2*, mapped to chromosome 9p13.3, have been identified in patients with AMDM. NPR-B, encoded by *NPR2*, contains four functional domains: an extracellular ligand-binding domain (ECD), a transmembrane domain, an intracellular kinase homology domain, and a guanylyl cyclase domain at the C-terminus (Potter and Hunter, 2001). Physiologically active NPR-B is a homodimer that catalyzes the formation of cGMP from GTP upon binding its ligand, C-type natriuretic peptide (CNP). The CNP/NPR2 signaling pathway is crucial for endochondral ossification, functioning to promote cartilage homeostasis and the proliferation and differentiation of osteoblasts and osteoclasts (Langenickel et al., 2004).

*NPR2* mutations cause broad-spectrum phenotypic variability. All affected individuals with AMDM carry a homozygous or compound heterozygous loss-of-function mutation. Heterozygous loss-of-function mutations in *NPR2* are associated with idiopathic short stature without skeletal dysplasia. They are also found in individuals with disproportionate short stature with skeletal anomalies, similar to those observed in SHOX negative-Léri-Weill dyschondrosteosis. However, no individuals have presented with Madelung deformity (Hisado-Oliva et al., 2015). By contrast, gain-of-function mutations in *NPR2* cause tall stature with mild scoliosis or overgrowth syndrome (epiphyseal chondrodysplasia, Miura type).

Here, we report two other AMDM cases of Chinese origin caused by compound heterozygous or homozygous loss-of-function mutations in *NPR2*, identified through whole-exome sequencing analysis. We evaluated the genotype-phenotype correlations in these patients and showed that the two novel mutations resulted in loss of function of *NPR2* based on structural and transcript analyses. Recombinant growth hormone (rhGH) treatment was administered to a 40-month-old affected child for more than 1 year.

## 2 Materials and Methods

### 2.1 Patients

The two patients and their family members provided written informed consent to participate in this study, and the study was approved by the Ethics Committee of Scientific Research and Clinical Trial of the First Affiliated Hospital of Zhengzhou University (approval no.2019-KY-401). Clinical information was extracted from medical records. All patients fulfilled the following diagnostic criteria: disproportional short stature, defined as a sitting height to height ratio greater than 2 standard deviation scores (SDSs) above the mean for the corresponding age and sex. Conventional laboratory tests could not explain the etiology of short stature. Laboratory examinations of patient 1 after rhGH therapy were performed before and after treatment, including serum alkaline phosphatase (AP), insulin-like growth factor (IGF)-1, total procollagen type 1 amino-terminal propeptide (P1NP), β-crosslaps, and osteocalein.

### 2.2 Whole-Exome sequencing and Targeted Next-Generation Sequencing

Genomic DNA was isolated from the peripheral blood of the probands of the two families using a DNA extraction kit (Omega, CA, United States). For patient 1, proband-only WES was performed and enriched for exonic sequences using an Agilent SureSelect XT Human All Exon 50 Mb kit (Santa Clara, CA, United States). For patient 2, targeted NGS using a genetic skeletal disease panel (including 225 genes; Supplementary Table S1) was performed by a commercial company (MyGenostics, Inc., Beijing, China). The quality of the library was assessed using Qubit 4.0 (Thermo Fisher Scientific Inc., USA). Paired-end sequencing was performed using an Illumina sequencing platform (Illumina, San Diego, CA, United States). After sequencing, data processing and variant annotation were performed using standard analyses (Li et al., 2021). High-quality reads were mapped to the human reference genome GRC37/hg19. Small variants were identified using Genome Analysis Toolkit version 3.8 (McKenna et al., 2010). For recessive model analyses, variants with a minor allele frequency of less than 0.01 in dbSNP138, 1000 Genomes, ExAC, and gnomAD databases were selected. Exonic and splice-site variants of 225 skeletal dysplasia related genes were collected for further analyses. The pathogenicity of variations was analyzed according to American College of Medical Genetics and Genomics (ACMG) guidelines (Richards et al., 2015).

### 2.3 Sanger Sequencing

Variants in *NPR2* were confirmed by Sanger sequencing, and paired primers were designed using Genetool software (Supplementary Table S2). Polymerase chain reaction (PCR) amplification with each primer set was carried out, and PCR products were sequenced on a 3130xl Genetic Analyzer (Applied Biosystems, Foster City, CA, United States). The data were analyzed using Chromas (Techne).

### 2.4 Reverse Transcription-PCR

Whole-blood RNA was extracted using TRIzol (Invitrogen, Carlsbad, CA, United States) and reverse transcribed using a HiScript II 1st Strand cDNA Synthesis Kit (Vazyme Biotech Co.,Ltd., Nan jing, China). cDNA sequences of *NPR2* from exons 15–22 were amplified by PCR, subcloned using a KOD FX Polymerase Kit (TOYOBO), and Sanger sequenced.

### 2.5 Bioinformatics Analysis

The pathogenicity of the identified variants was assessed using Varcards (http://varcards.biols.ac.cn/) and Pubvar (https://www.pubvar.com/) using various tools, including the Rare Exome Variant Ensemble Learner, Sorting Intolerant from Tolerant, Likelihood Ratio Test, Combined Annotation Dependent Depletion, Polymorphism Phenotyping V2, and MutationTaster. Genetic variants in *NPR2* were retrieved from the ClinVar and professional HGMD databases. Alignments were made of *NPR2* from *Homo sapiens*, mice, rhesus monkeys, dogs, and elephants to identify amino acid conservation at novel missense mutation sites.

### 2.6 Homology Modeling and Molecular Dynamics Simulation

The ECD of HsNPR2 was modeled as follows. First, the template for modeling was retrieved from the Protein Database (PDB; http://www.rcsb.org); using the Basic Local Alignment Search Tool (BLAST). The wild-type ECD (ECD^wt^) of HsNPR2 (amino acids:17–421) was modeled on the NPR-A crystal structure (PDB entry 1DP4). Subsequent modeling was performed using the MODELLER program, and discrete optimized protein energy (DOPE) scores in terms of spatial restraints of amino acids were ranked to assess model quality. On the basis of DOPE scores, the best quality model was selected and the model quality was then monitored using the SAVES (services.mbi.ucla.edu/SAVES/) and ProSAweb validation servers (https://prosa.services.came.sbg.ac.at/prosa.php) (Ramachandran et al., 1963). The homomeric structure of ECD^wt^ was generated using the GalaxyWeb server (http://galaxy.seoklab.org/cgi-bin/submit. cgi?type = HOMOMER). The mutant ECD (ECD^371Q^) was generated by inputting the corresponding mutant amino acid sequences of ECD in the same analysis process as described above.

Homodimers of ECD^wt^/ECD^371Q^ were subjected to MD simulations for structural refinement. MD simulations were performed with the TIP3P water model using the Gromacs 2021 package, and the topology of the protein structure was generated by CHARMm36ff parameterization (Irfanullah et al., 2018). Additionally, 0.1 M NaCl was added to neutralize the system, and energy minimization was performed using the steepest descent algorithm (at a maximum force of 10 kJ/mol) to avoid steric clash. Each system was heated at 300 K using the V-rescale method (Bussi et al., 2007), and pressure was equilibrated at 1.0 bar using a Parrinello-Rahman barostat (Parrinello and Rahman, 1981). Finally, the MD simulation for each homodimer was run for 100 ns. The coordinates of the refined structures were extracted from the final trajectory frame. The MD refined models of homology ECD were validated using the SAVES and ProSAweb validation servers and were exported into Discovery Studio 2016 for further analyses. Comparative analyses were performed to determine the structural variations between ECD^wt^ and ECD^371Q^.

### 2.7 Ligand Preparation and Molecular Docking

The CNP model was extracted from the NPR-C crystal (PDB entry 1jdp) using PyMOL (Rootsi et al., 2004). We used the HPEPDOCK server to perform molecular docking of CNP with the MD refined model of homology ECD^wt^ and ECD^371Q^ based on the hierarchical algorithm (Zhou et al., 2018). The HPEPDOCK webserver is available at http://huanglab.phys.hust.edu.cn/hpepdock/.

## 3 Results

### 3.1 Clinical Features

**FIGURE 1:**
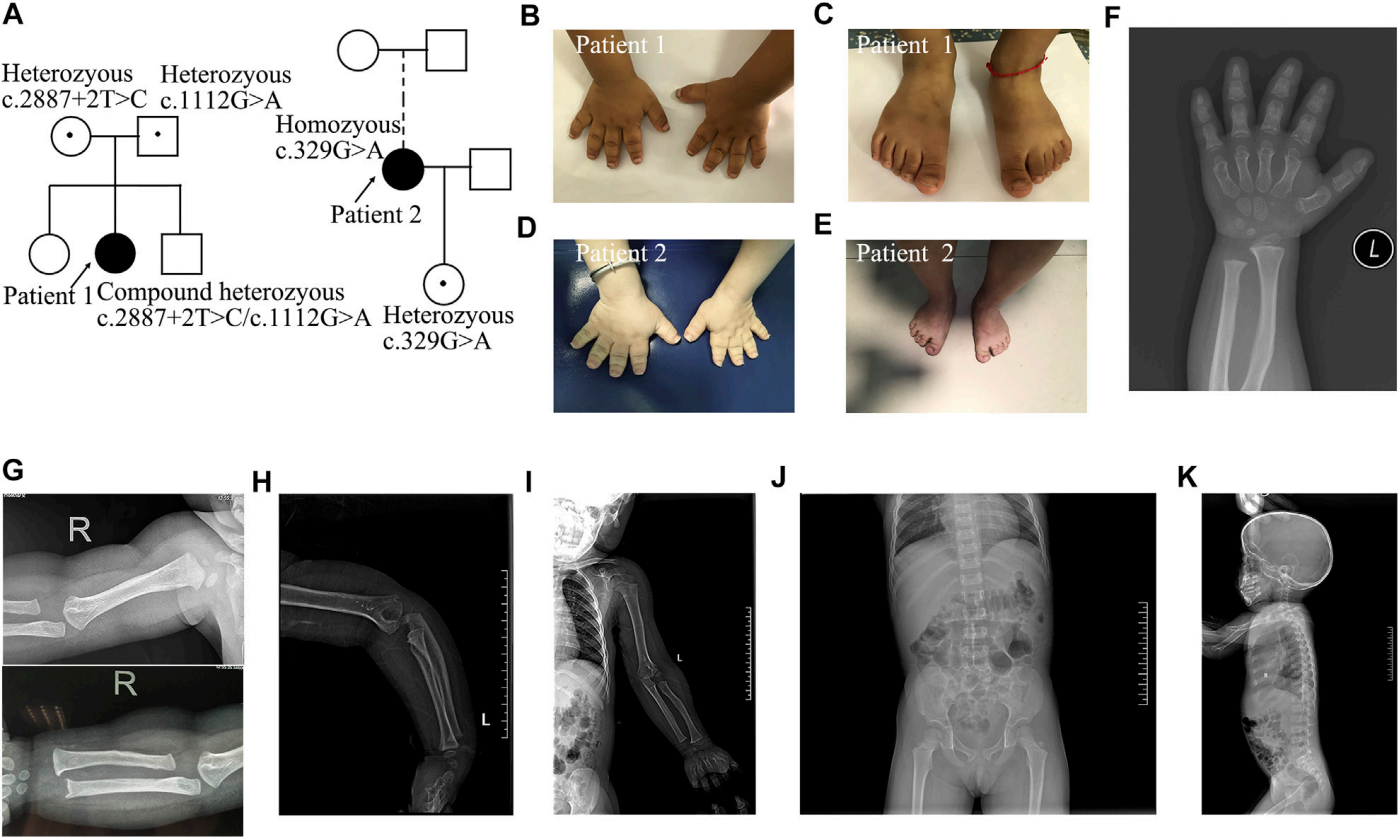
Pedigrees and images of two patients with Acromesomelic Dysplasia Maroteaux Type. **(A)** Pedigree of two families containing patient 1 and 2; **(B,C)** small hands and feet in patient 1; **(D,E)** patient 2 with loose and redundant skin on fingers and feet; **(F)** short and dysplastic metacarpals and phalanges in patient 1; **(G-I)** The humerus and radius are curved, besides, osteophyte existed in both distal ulna and lateral epicondylar humeri in patient 1. There was no dislocation of the radial head. There were no missing or fused bones; **(J,K)** Mild scoliosis and irregular anterior wedging of vertebral bodies in patient 1.

Patient 1 was a girl who presented with short stature and small stubby fingers (Figure 1; Supplementary Table S3), which were noticed at 6 months of age. She was born by cesarean section after 37 weeks of gestation as the second child of healthy, nonconsanguineous parents. Her birth weight was 2,800 g, and her birth length was 50 cm. At 3 years and 4 months of age, her sitting height to standing height ratio was 0.575. Her psychomotor development, intelligence, and cognitive development were normal. Physical examination revealed noticeably short upper and lower extremities, brachydactylic fingers and toes (Figures 1B,C), apparent prominent forehead, long face, short and broad nose, pronounced shortening, and slightly bowed forearm. The radiological manifestations were compatible with AMDM (Figures 1F–K). Her parents and siblings had proportionate bodies without small hands, feet, or extremities.

Patient 2 was a 31-year-old woman who presented with disproportionate acromesomelic dysplasia (Figure 1; Supplementary Table S3). Her arm span was 108 cm, and her arm span to height ratio was 0.86. Her sitting height was 75.7 cm, with a sitting to standing height ratio of 0.6. Her upper and lower segments were measured at 59.5 and 66 cm, respectively, with an upper to lower segment ratio of 0.9. Her head circumference was 52.5 cm. Her foot span was 17 cm, and her hand span was 10 cm. Prominent forehead, long face, low-set ears, high-arched palate, short neck, torticollis with normal forward and backward head movement, uneven shoulder with the left side relatively higher, bilateral short broad thumbs and toes, large halluces, and loose and redundant skin on the hands were noted (Figures 1D,E). She did not show clinical evidence of Madelung deformity, scoliosis, brachydactyly, and clinodactyly. She refused further imaging examination. Her menarche occurred at 15 years of age. Her 5-year 11-month-old daughter presented with proportionate dwarfism. Both the proband and her daughter had normal intelligence, hearing, and speech.

### 3.2 rhGH Treatment and Related Laboratory Measurements in Patient 1

**FIGURE 2:**
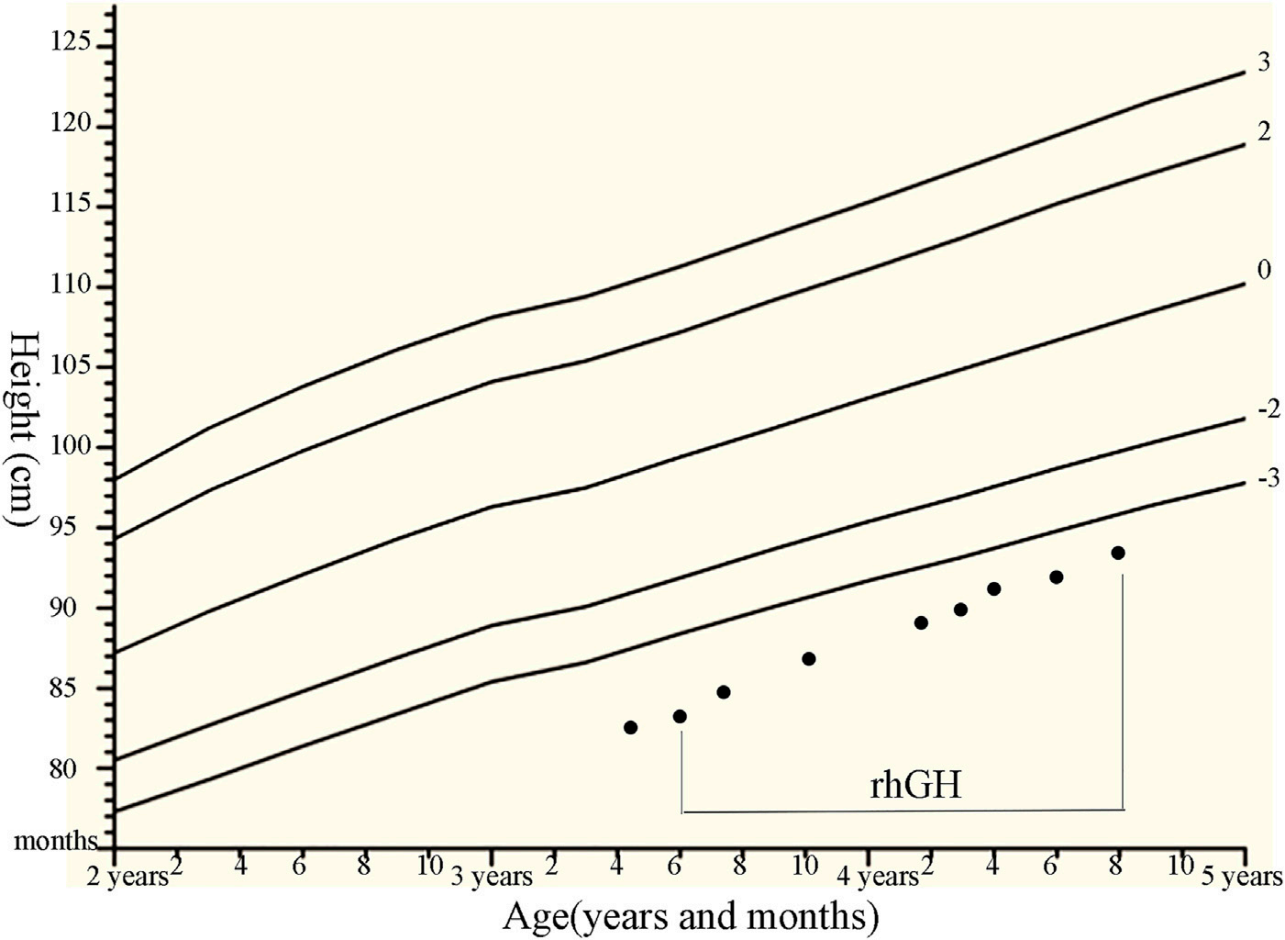
Growth chart of the patients with compound heterozygous NPR2 mutations (c.1112G>A and c.2887+2T>C) with the rhGH therapies. We plotted the height on standardized growth charts for Chinese children and adolescents aged 0–18 years (Li et al., 2009), and evaluate rhGH treatment efficacy for our patient.

Patient 1 was treated with rhGH (from 0.35 mg/kg/week at initial treatment to 0.47 mg/kg/week, using daily subcutaneous injections). Subsequently, her growth velocity improved by 10.4 cm after 15 months of rhGH treatment, with a height gain of +0.6 SDS (Figure 2). There were no considerable adverse effects and no clinical deterioration of skeletal deformities. The association of height gain with steady increases in serum IGF-1 and P1NP levels following initiation of GH treatment and dose increases was observed in the patient, but no obvious changes in serum AP, N-MID, and β-crosslap levels were observed during rhGH treatment (Supplementary Table S4).

### 3.3 Genetic Analysis

**FIGURE 3:**
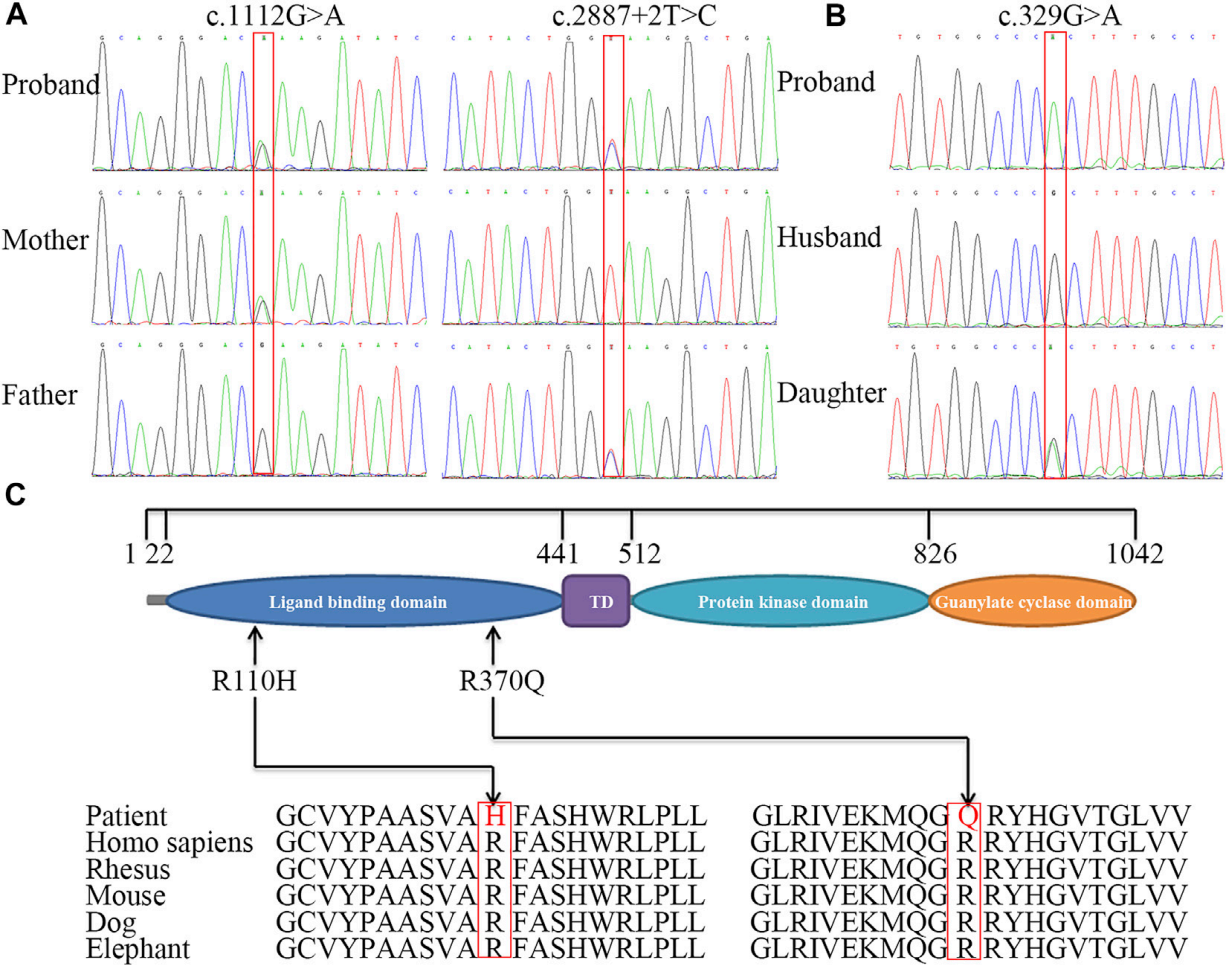
Sequence analysis of *NPR2* gene and conservation analysis of missense mutations. Partial DNA sequence of three variants including c.1112G>A and splice donor site variant identified in family 1, mother and father are carriers for above mentioned mutation **(A)**, and missense homozygous mutation c.329G>A identified in patient 2, her daughter is carrier for the same mutation **(B)**. The structure of *NPR2* and the position of mutations **(C)**. The different geometric shapes with different colors on amino acid (aa) sequence denote the four distinct functional regions respectively. Its amino acid substitution in two patients were both highly conservative substitution.

Using whole-exome sequencing of patient 1, we identified two single nucleotide variants in *NPR2* (NM_003995.3): c.1112G>A p.(Arg371Gln) and c.2887+2T>C (Figure 3A; Supplementary Figures S1, S2). Sanger sequencing showed that the c.1112G>A variant was inherited from the mother, whereas the c.2887+2T>C variant was inherited from the father. Analysis of sequencing data from patient 2 revealed a homozygous missense mutation, c.329G>A p.(Arg110His), in exon 1 of *NPR2* (Figure 3B; Supplementary Figure S3). Sanger sequencing showed that her daughter was heterozygous for the mutation. Three variants were respectively classified as “likely pathogenic variant” (PM1+PM2_supporting + PM3+PP3+PP4, PVS1+PM2_supporting + PP3+PP4 and PS1+ PM2_supporting + PP3+PP4).

Patient 2 was adopted and her biological paternal samples were not available. The results showed that the two families segregated AMDM in an autosomal recessive manner. The missense variants [c.1112G>A p.(Arg371Gln) and c.329G>A p.(Arg110His)] were predicted to be pathogenic or deleterious using various online tools (Supplementary Table S5). The amino acids Arg371 and Arg110 were completely conserved among mammals, including rhesus monkeys, mice, dogs, and elephants (Figure 3C).

### 3.4 Multiple Forms of Aberrant Splicing for the Splice Mutation c.2887+2T>C

**FIGURE 4:**
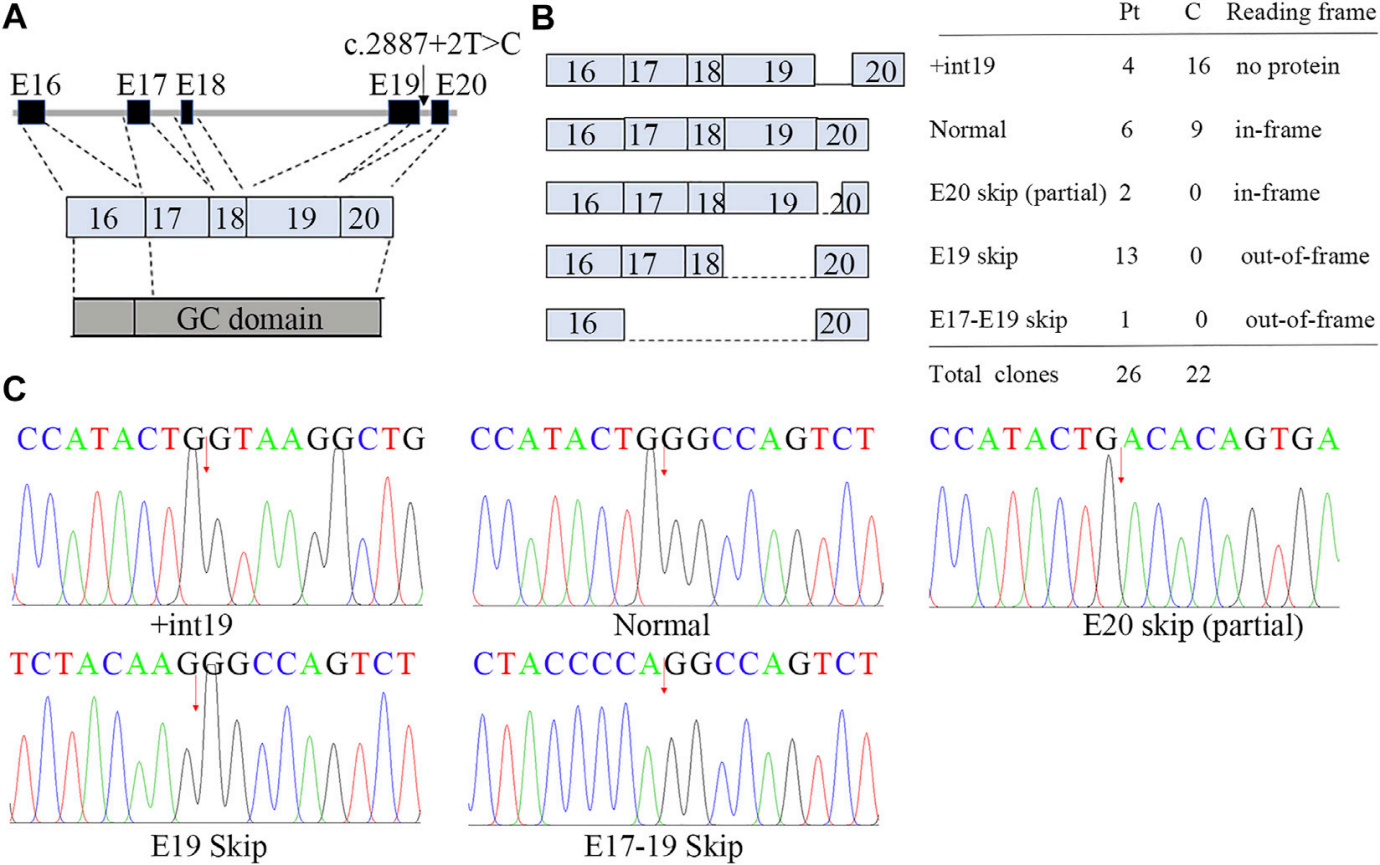
Aberrant splicing of c.2887+2T>C. **(A)** Genomic architecture of *NPR2* gene through exons 16 to 20. **(B)** Subcloning of the RT-PCR product demonstrated multiple forms of aberrant splicing. The table shows the number of clones. **(C)** cDNA sequencing of breakpoint in *NPR2*. The possible breakpoints are indicated with arrows.

We showed that the RNA of patient 1 was aberrantly spliced and lacked partial exon 20 (c.2888-2944), exon 19 (c.2713-2887), or exons 17–19 (c.2520-2877; Figure 4; Supplementary Figure S4). The transcript generated from the skipping of exon 19 was out-of-frame and was predicted to produce a truncated protein of about 101 kDa [p.(Asp901GlyfsX9)]. By contrast, the transcript generated from the partial skipping of exon 20 lacked 19 amino acids [p.(Gly963 to Gly981)]. The transcript generated from skipping of exons 17–19 was also out-of-frame and was translated into a truncated protein [p.(His840GlnfsX31)].

### 3.5 Structural and Functional Analysis of the Novel Missense Mutation p.(Arg371Gln)

**FIGURE 5:**
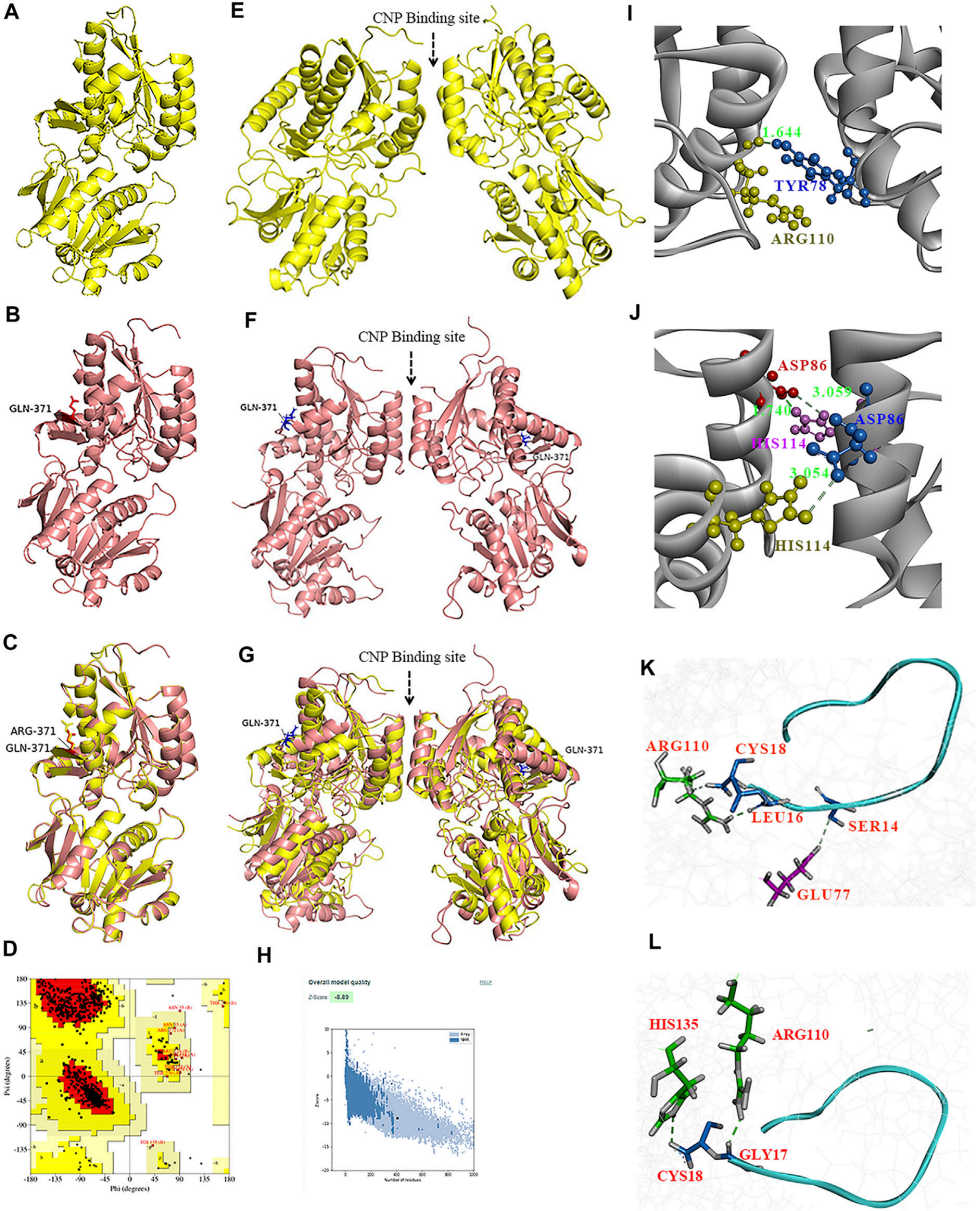
Homology modeling of the wild type and mutant ECD of HsNPR2. A three-dimensional model of the monomer of ECD^wt^ **(A)**, monomer of ECD^371Q^ **(B)**, and the graphic superimposition between the monomer of ECD^wt^ and ECD^371Q^ **(C)**. Validation of the homology ECD domain model of HsNPR2. Residues distribution of the ECD **(D)** in the correspondent regions of Ramachandran plot. Z-score validation of the ECD **(E)**. The MD refined homo-dimer of ECD^wt^ **(F)**, the MD refined homo-dimer of ECD^371Q^ **(G)** and the graphic superimposition of the MD refined homology between ECD^wt^ and ECD^371Q^ **(H)**. Comparing to ECD^wt^, the binding between CNP and the MD refined homodimer of ECD^371Q^ is arrow-pointed in panel. Structural and molecular analyses suggest mutant ECD of NPR2 constricts the CNP binding site due to enhanced polar interactions in the homodimer **(I,J)**. One hydrogen bond formation between Try78 and Arg110 in the ECD^wt^ **(I)** and three hydrogen bonds formation between D86 and H114 **(J)**. By comparison to ECD^wt^ **(K)** of NPR2, lessened molecular interactions observed on the ligand CNP to the homodimer of and ECD^mt^ **(L)**.

To test whether the p.(Arg371Gln) missense mutation influenced the binding capacity of CNP to NPR-B, we derived three-dimensional homology models of the ECD of NPR-B (Figure 5). A PDB library BLAST search was performed to identify the appropriate template for the NPR-B ECD domain. PDB entry 1DP4 (59.6% similarity with ECD) was selected as the template for homology modeling. The model having the lowest DOPE score (−49087.96) was selected for further exploration. After stereochemical validation and Z-score analysis, minor deviation around the mutation site was intuitively observed by superimposition of the monomer model of ECD^wt^ and ECD^371Q^ (Figures 5A–C). The homomeric structures of ECD, produced by the GalaxyWeb server, were subjected to MD simulation for initial structural refinement (Supplementary Figures S5, S6). The last frame of the trajectory was selected for further analysis of the coordinates of the MD refined homology ECD domain. The stereochemical validation of the MD refined homology ECD revealed that approximately 89.4% of residues occupied the most favored region in the Ramachandran plot (Figure 5D). Z-score (−8.5) analysis using the ProSAweb server demonstrated that the quality was sufficient for subsequent analyses (Figure 5H). Upon superimposition between the homodimers of ECD^wt^ and ECD^371Q^, we observed that the CNP binding site of ECD^371Q^ was constricted and may result in a reduction in CNP binding (Figures 5E–G). In addition, analysis of the molecular interactions between homodimers indicated one hydrogen bond interaction between Try78 and Arg110 in ECD^wt^, three hydrogen bond interactions between Asp86 and His114, and more π-cation interactions in ECD^371Q^ (Figures 5I,J; Table 1).

**TABLE 1:** Molecular interactions between the MD refined ECD homo-dimer of NPR2.

MD refined ECD homo-dimer of NPR2	Hydrogen bonds		π-cation interactions	
	Chain A	Chain B	Chain A	Chain B
Wild type	R110	Y78	R110	Y78
			H135	Y78
			F111	L82
			H114	L79
			H114	L82
			L82	F111
			V85	F111
			L82	H114
Mutant (R371Q)	D86	H114	H110	Y78
	H114	D86	Y78	R110
	D86	H114	L82	H114
			Y78	R110
			F111	L82
			F111	V85
			H114	L82
			V85	F111
			L71	H114

Amino acid residues are denoted by single letter symbols i.e.D = Asp, E = Glu, F=Phe, H=His, L = Leu,R = Arg, V=Val, Y = Tyr.

Next, we performed molecular docking studies between CNP and homodimers (ECD^wt^ and ECD^371Q^, Figures 5K,L) using the HPEPDOCK server. Compared with the wild-type homodimers of NPR-B, molecular interactions of CNP with mutated homodimers of NPR-B were reduced by one hydrogen bond interaction and one salt bridge interaction (Table 2), suggesting that ECD^371Q^ homodimers of NPR-B weakened the binding of CNP to the mutated homodimers of NPR-B.

**TABLE 2:** Molecular interactions between CNP and the MD refined ECD homo-dimer of NPR2.

MD refined ECD homo-dimer of NPR2	Hydrogen bonds			Salt bridges interactions		
	CNP	Chain A	Chain B	CNP	Chain A	Chain B
Wild type	L16	R110		K6	E77	
	C18	R110		R9		D134
	S14		E77			
Mutant (R371Q)	G17	R110		R9		E185
	C18	H135				

Amino acid residues are denoted by single letter symbols i.e C=Cys, D = Asp, E = Glu, G = Gly, H=His, L = Leu, K = Lys, R = Arg, S=Ser.

## 4 Discussion

In the current study, we performed clinical and molecular evaluations of two Chinese patients with AMDM exhibiting the phenotype of short stature and characteristic shortening of the middle and distal segments of the limbs. For Chinese patients, only one patient with AMDM has been reported. WES analysis revealed the novel compound heterozygous mutations c.2887+2T>C and c.1112G>A p.(Arg371Gln) in *NPR2* in patient 1. In addition, a homozygous missense mutation c.329G>A p.(Arg110His) was found in patient 2, which was recently reported (Simsek-Kiper et al., 2021). The pathogenicity of the two novel mutations (c.2887+2T>C, c.1112G>A) was confirmed by segregation analysis, transcript analysis, and in silico analysis. Heterozygous *NPR2* mutations with dominant-negative effects have been first observed in patients with idiopathic short stature (Bartels et al., 2004). Our finding that heterozygous carriers of variants (c.1112G>A, c.2887+2T>C, c.329G>A) also had a subtler proportionate short stature suggested that heterozygous mutations caused haploinsufficiency or topology modification of *NPR2*, which resulted in the loss of height potential.

Most AMDM-related *NPR2* mutations are hypothesized to cause disease by impairing trafficking to the plasma membrane, altering CNP ligand binding affinity, or inhibiting the activity of NPR-B. Recently, Irfanullah et al. suggested that the missense mutation p.(Leu314Arg) allosterically affects the binding of NPR-B homodimer to CNP (Irfanullah et al., 2018). The novel missense variant p.(Arg371Gln) identified in patient 1 was positioned in the CNP-ligand binding domain. In silico modeling analysis showed that inter-residual molecular interactions of the mutant structure were enhanced compared with that in the wild-type owing to enhanced hydrogen bond formation between Asp86 of one monomer and His114 of the other monomer. Our modeling results were consistent with the speculation that the mutation disrupted the CNP binding site in the extracellular domain as a result of the global allosteric effects of the homodimer. The splice mutation c.2887+2T>C identified in patient 1 was located in intron 19 of *NPR2*. An RNA/cDNA study was performed to analyze the consequences of c.2887+2T>C, which resulted in three aberrantly spliced transcripts in patient 1. The main transcript, lacking exon 19, was predicted to produce a truncated protein lacking a large proportion of the guanylate cyclase domain. Therefore, this mutation is likely to cause disease by suppressing the activity of NPR-B. Subcellular localization studies have shown that the mutation p.(Arg110Cys) in *NPR2*, identified in a Japanese family showing the phenotype of short stature, is defective in cellular trafficking from the endoplasmic reticulum to the Golgi apparatus (Jacob et al., 2018). We hypothesize that the reported missense mutation p.(Arg110His) may cause AMDM by impairing trafficking to the plasma membrane.

Evaluation of typical facial features and radiological data for skeletal involvement will be beneficial to the clinical diagnosis of AMDM. Patient 1 had more striking skeletal dysplasia in the middle and distal extremities than in the trunk. Patient 2 and the reported 9-year-old boy carried the same homozygous mutation c.329G>A in *NPR2* (Supplementary Table S3). The above-mentioned boy had mild obstructive sleep apnea, whereas patient 2 did not. No radiological data were available for patient 2. Current information demonstrated that morphological changes in vertebral bodies with age may increase the risk for subsequent development of spinal stenosis (Ain et al., 2019) and obstructive sleep apnea (Huang et al., 2012; Simsek-Kiper et al., 2021), which will seriously influence patient quality of life and should be considered during follow-up. Tricuspid regurgitation was found in patient 1 in our study and mitral valve insufficiency in a 54-year-old female patient and the above-mentioned boy with AMDM (Simsek-Kiper et al., 2021). The co-occurrence of heart valve diseases may be because of the high parental consanguinity rate, which may contribute to heart valve diseases probably related to other gene mutations (Simsek-Kiper et al., 2021). Although NPR-B has already been shown to mediate the effects of aortic valve development and disease in mice (Blaser et al., 2018), the relationship between *NPR2* and heart valve diseases is unclear and should be explored in the future. Patient 2 had a 6-year-old daughter who was conceived without the use of any type of assisted reproductive technology. To date, most reported cases have been in children. No reports have described female fertility in adults affected with AMDM; however, a few other pedigrees have also shown normal fertility in men with the AMDM phenotype (Irfanullah et al., 2018). A distinct *NPR2*-knockout mouse model harboring a 4-bp deletion in exon 3 exhibited dwarfism and female sterility with normal pituitary and uterine function (Geister et al., 2013). The precise contribution of NPR-B to human reproduction is not yet clear.

Few reports have described long-term data in patients with AMDM receiving rhGH treatment. To date, there had been three patients with AMDM who received rhGH treatment, reported in two different studies; however, the therapeutic effects of rhGH treatment were controversial. Arya et al. (2020) found that the final heights of the two patients with AMDM (130.5 and 134 cm, respectively) were significantly greater than the average reported final height (110–120 cm) of patients with AMDM after long-term rhGH treatment (0.525–0.7 mg/kg/week, 8 years). Olney, (2006) have suggested that one patient with AMDM showed poor responses to high-dose rhGH treatment (0.35 mg/kg/week, 1.5–5.5 years of age) owing to resistance to the effects of rhGH. In this study, rhGH therapy improved the linear growth of proband 1 after high-dose GH treatment (0.35 mg/kg/week, 3.4–5 years of age). In these three GH-responsive patients with AMDM including those in the current study, IGF-1 levels were within or below the lower limit of the normal range but increased steadily during rhGH treatment. In addition, strikingly lower IGF-1 levels were found in NPR2-knockout mice than in wild-type littermates (Olney, 2006). The mechanism described below may partly explain how rhGH treatment improves bone growth disturbance in patients with AMDM. rhGH restores plasma IGF-1 levels (Binder et al., 2005; Wagner et al., 2021) and sensitivity to IGF-I in local distinct cell lines or to its autocrine/paracrine action (Lebl et al., 2001). IGF-1 further inhibits the p38 mitogen-activated protein kinase cascade (Studer et al., 2004) and promotes high extracellular signal-regulated kinase/p38 activity ratios favoring chondrocyte proliferation (Hutchison, 2012). Notably, rhGH treatment has already been shown to have positive effects on height improvement in AMDM and many other types of skeletal dysplasia (Camtosun et al., 2019; Levy-Shraga et al., 2020; Cetin et al., 2018; Sarafoglou et al., 2010; Utsumi et al., 2017). Additional studies are needed in other individuals with AMDM to determine whether rhGH treatment is effective.

To date, 71 pathogenic mutations in *NPR2* have been shown to be associated with AMDM based on an inclusive review of the literature (Mustafa et al., 2020; Simsek-Kiper et al., 2021) (including the two current cases) and two databases (Human Gene Mutation Database and Leiden Open Variation Database; Supplementary Table S6; Supplementary Figure S7), including 39 missense mutations, 15 nonsense mutations, six splicing mutations, 10 deletions, and one insertion. Biallelic mutations in *NPR2* that underlie AMDM are found throughout the entire length, except for in exons 9 and 18. Exons 1 (14/71, 19.72%), 6 (6/71, 8.45%), and 19 (9/71, 12.68%) had higher point mutation frequencies than any of the other exons. Biallelic mutations in *NPR2* that underlie AMDM are found throughout the entire length, except for in exons 9 and 18. These identified variants were mainly located in the extracellular CNP-binding domain (47.9%) and intracellular guanylyl cyclase domain (28.2%). Almost all families show unique *NPR2* variants, suggesting a high proportion of segregation through families. It is difficult to identify the genotype-phenotype correlations of distinct mutations in different functional regions of NPR-B or even in the same functional region.

There were some limitations to our study. First, because the biological parents of patient 2 were unavailable, clinical phenotypes and segregation analysis could not be performed in this study. Second, owing to the absence of previous clinical records, we could not describe the developmental data of patient 2 in this study. Third, it may still be important to investigate the molecular effect of these mutations in *NPR2 in vitro* in the future. Therefore, further studies and randomized controlled trials with more patients are needed to confirm the effects of rhGH therapy on final height in patients with AMDM.

In conclusion, clinical and molecular evaluations produced three different variants in *NPR2* in two families from China, manifesting the variable clinical features of AMDM. The identified novel mutations, c.2887+2T>C and p. Arg371Gln, exerted dominant-negative effects, reducing the activity of NPR-B and the binding affinity of NPR-B to CNP. Our findings indicated that the two novel mutations were loss-of-function mutations. Relatively short-term high-dose rhGH treatment significantly increased the height SDS of patient 1. Further studies and randomized controlled trials with more patients are needed to confirm the effects of rhGH therapy on final height in patients with AMDM.

## Data Availability

The datasets for this article are not publicly available due to concerns regarding participant/patient anonymity. Requests to access the datasets should be directed to the corresponding authors.
