# Supplementary material for: Novel Loss-of-Function Mutations in NPR2 Cause Acromesomelic Dysplasia, Maroteaux Type
Source: Front Genet. 2022 Mar 16;13:823861. doi: 10.3389/fgene.2022.823861 (PMC8967736; doi:10.3389/fgene.2022.823861)
Supplement: Supplementary file 2 [file DataSheet2.docx]

**Supplementary Table 1. 225 genes of a genetic skeletal disease panel.**

| *ACAN* | *ACP5* | *ACVR1* | *ADAMTSL2* | *AGA* | *AGPS* | *ALPL* |
| --- | --- | --- | --- | --- | --- | --- |
| *ALX1* | *ALX3* | *ALX4* | *ANKH* | *ANO5* | *ANTXR2* | *ARSB* |
| *ARSE* | *B4GALT7* | *BMP1* | *BMP2* | *BMPER* | *BMPR1B* | *CA2* |
| *CANT1* | *CASR* | *CC2D2A* | *CDH3* | *CEP290* | *CHST14* | *CHST3* |
| *CLCN5* | *CLCN7* | *COG1* | *COL10A1* | *COL11A1* | *COL11A2* | *COL1A1* |
| *COL1A2* | *COL2A1* | *COL9A1* | *COL9A2* | *COL9A3* | *COMP* | *CREBBP* |
| *CRTAP* | *CTSA* | *CTSK* | *CUL7* | *DDR2* | *DHCR24* | *DHODH* |
| *DLL3* | *DLX3* | *DMP1* | *DYM* | *DYNC2H1* | *EBP* | *EFNB1* |
| *EIF2AK3* | *ENPP1* | *EP300* | *ESCO2* | *EVC* | *EVC2* | *EXT1* |
| *EXT2* | *FAM20C* | *FAM58A* | *FBLN1* | *FBN1* | *FBN2* | *FBXW4* |
| *FERMT3* | *FGF10* | *FGF23* | *FGF9* | *FGFR1* | *FGFR2* | *FGFR3* |
| *FKBP10* | *FLNA* | *FLNB* | *FMN1* | *FUCA1* | *GALNS* | *GALNT3* |
| *GDF5* | *GDF6* | *GJA1* | *GLB1* | *GLI3* | *GNAS* | *GNPTAB* |
| *GNPTG* | *GNS* | *GORAB* | *GPC6* | *GREM1* | *GUSB* | *HDAC4* |
| *HES7* | *HGSNAT* | *HOXA11* | *HOXA13* | *HOXD13* | *HPGD* | *HSPG2* |
| *ICK* | *IDS* | *IDUA* | *IFITM5* | *IFT122* | *IFT80* | *IHH* |
| *IL1RN* | *LBR* | *LEMD3* | *LFNG* | *LIFR* | *LMBR1* | *LMNA* |
| *LMX1B* | *LPIN2* | *LRP4* | *LRP5* | *MAN2B1* | *MAN2C1* | *MATN3* |
| *MESP2* | *MGP* | *MKS1* | *MMP13* | *MMP2* | *MMP9* | *MNX1* |
| *MSX2* | *MYCN* | *NAGLU* | *NEK1* | *NEU1* | *NF1* | *NFIX* |
| *NIPBL* | *NKX3-2* | *NLRP3* | *NOG* | *NPPC* | *NPR2* | *NSD1* |
| *NSDHL* | *OBSL1* | *OFD1* | *OSTM1* | *P3H1* | *PAPSS2* | *PCNT* |
| *PEX7* | *PHEX* | *PIGV* | *PITX1* | *PLEKHM1* | *PLOD2* | *POLR1C* |
| *POLR1D* | *POR* | *PPIB* | *PTH1R* | *PTHLH* | *PTPN11* | *RAB23* |
| *RASGRP2* | *RECQL4* | *ROR2* | *RPGRIP1L* | *RUNX2* | *SALL1* | *SALL4* |
| *SBDS* | *SERPINF1* | *SERPINH1* | *SH3BP2* | *SH3PXD2B* | *SHH* | *SHOX* |
| *SLC17A5* | *SLC26A2* | *SLC34A3* | *SLC35D1* | *SLC39A13* | *SLCO5A1* | *SMARCAL1* |
| *SOST* | *SOX9* | *SP7* | *SULF1* | *SUMF1* | *TBCE* | *TBX15* |
| *TBX3* | *TBX4* | *TBX5* | *TBXAS1* | *TCIRG1* | *TCOF1* | *TGFB1* |
| *TGFBR1* | *TGFBR2* | *THPO* | *TMEM38B* | *TMEM67* | *TNFRSF11A* | *TNFRSF11B* |
| *TNFSF11* | *TP63* | *TRAPPC2* | *TREM2* | *TRIP11* | *TRPS1* | *TRPV4* |
| *TWIST1* | *TYROBP* | *WDR35* | *WISP3* | *WNT1* | *WNT3* | *WNT7A* |
| *ZMPSTE24* |  |  |  |  |  |  |

**Supplementary Table 2. Primer sequence in the study.**

| Primer Name | Sequence (5'-3') |
| --- | --- |
| *NPR2*-E1F | GCCAGAACACAACCTGAGCTA |
| *NPR2*-E1R | TGCGAACCAGGGTACGATAA |
| *NPR2*-E4F | CTTGCAGGACATTTGGAGAGTACT |
| *NPR2*-E4R | TTCCCCGATCTTGAGGTGTG |
| *NPR2*-E19F | CTCCCCTAGACTCAGGACCA |
| *NPR2*-E19R | GGCATCTTCAGGCCAACAAC |
| RT-F | CTCAGCCCCAAAGAGATTGTCC |
| RT-R | CCAGGTACCAGCAGCAGAAGACTA |

**Supplementary Table 3.** **Clinical and molecular features of AMDM patients with *NPR2* variants**

| Family ID | 1 | 2 | 3 |
| --- | --- | --- | --- |
| Patient ID | II:1 | I:1 | 4 |
| Reference | this study | this study | Simsek-Kiper et al., 2021 |
| Variant (NM_003995) | c.1112G>A p.(Arg371Gln) in exon 4 ;c.2887+2T>C in intron 19 | c.329G>A p.(Arg110His) in exon 1 | c.329G>A p.(Arg110His) in exon 1 |
| Variant status | Compound heterozygous | Homozygous | Homozygous |
| Parental *NPR2* variant status | Heterozygous | Not known | Heterozygous |
| Consanguinity | ─ | Not known | First cousin marriage |
| Gender | Female | Female | Male |
| Age at molecular diagnosis, years | 3 | 31 | 9 |
| Height (cm/SDS) | 82.6/-4.67 | 125.5/-6.5 | E |
| Weight(Kg/SDS) | 11/-2.58 | 38/-2.18 | E |
| Typical facial features | Yes | Yes | Yes |
| Typical skeletal and radiographic findings | Yes | ─ | Yes |
| Motor developmental delay | No | No | No |
| other clinical findings | tricuspid regurgitation | uneven shoulder with the left side relatively higher | Mitral valve insuffieciency, adenoidectomy, bilateral single palmar crease, mild obstructive sleep apnea |
| Heterozygous carriers height, cm/SDS | her mother 147/−2.74; her father 168/−1.33 | her daughter 106.5/-2.20 | his mother 147/−2.74; his father 168/−1.33 |
| ─:not available; E: wrong description | | |  |

**Supplementary Table 4.** **Laboratory measurements of serum in patient 1.**

| patient 1 | AP (0–281 U/L) | IGF-1 (49–289 ng/mL) | P1NP (15.13–58.69 ng/mL) | β-crosslaps ( < 0.573 ng/mL) | N-MID (11–43 ng/mL) |
| --- | --- | --- | --- | --- | --- |
| Before GH treatment | 212 | 70 | 604.1 | 1.1 | 51.3 |
| After GH treatment |  |  |  |  |  |
| 4 months | ─ | 120 | 739.2 | 2.13 | 67.89 |
| 7 months | 215 | 151.9 | ─ | ─ | ─ |
| 10 months | ─ | 167.7 | ─ | ─ | ─ |
| 15 months | ─ | 201.3 | 768.9 | 1.57 | 54.49 |

AP:alkaline phosphatase;IGF-1: insulin-like growth factor-1;P1NP: total procollagen type 1 amino-terminal propeptide; N-MID:osteocalein; ─:not available.

**Supplementary Table 5. In-silico missense prediction showing the two variants of disease-causing.**

| **Algorithm** | **c.1112G>A** | **c.329G>A** |
| --- | --- | --- |
|  | **score/prediction** | **score/prediction** |
| REVEL | 0.597/Damaging | 0.709/Damaging |
| SIFT | 0.025/Damaging | 0.004/Damaging |
| Polyphen-2_HDIV | 0.994/Probably_damaging | 1.000/Probably_damaging |
| Polyphen-2_HVAR | 0.733/Possibly_damaging | 0.988/Probably_damaging |
| LRT | 0.003/Neutral | 0.000/Deleterious |
| Mutation Taster | 1.000/Disease_causing | 1.000/Disease_causing |
| M-CAP | 0.172/Damaging | 0.300/Damaging |
| CADD | 33/Damaging | 31/Damaging |
| DANN | 0.999/Damaging | 1.000/Damaging |
| FATHMM | 0.967/Damaging | -1.84/Damaging |
| Eigen | 0.633/Damaging | 0.594/Damaging |
| GenoCanyon | 1.000/Damaging | 1.000/Damaging |
| GERP++ | 5.69/Conserved | 3.93/Conserved |
| phylop | 7.437/Conserved | 4.372/Conserved |
| phastCons | 1.000/Conserved | 1.000/Conserved |
| Siphy | 15.914/Conserved | 13.630/Conserved |

**Supplementary Table 6. Mutation numbers of different types of NPR2 mutations related to AMDM in four functional domains.**

| Mutation type | Mutation number(n) | | | | | Percentage (%) | |  |
| --- | --- | --- | --- | --- | --- | --- | --- | --- |
|  | | extracellular ligand-  binding domain | transmembrane  region | intracellular kinase  homology domain | carboxyl-terminal  guanylyl cyclase domain | |  | |
| missense | | 21 | 0 | 7 | 11 | | 54.9% | |
| nonsense | | 6 | 2 | 4 | 3 | | 21.1% | |
| del/ins | | 6 | 1 | 0 | 4 | | 15.5% | |
| splice | | 1 | 1 | 2 | 2 | | 8.5% | |
| Percentage (%) | | 47.9% | 5.6% | 18.3% | 28.2% | | 100% | |

**Supplementary Figure 1. Screenshot of the exome alignment of c.1112G>A p. (Arg371Gln) in the patient 1 (the black box).**


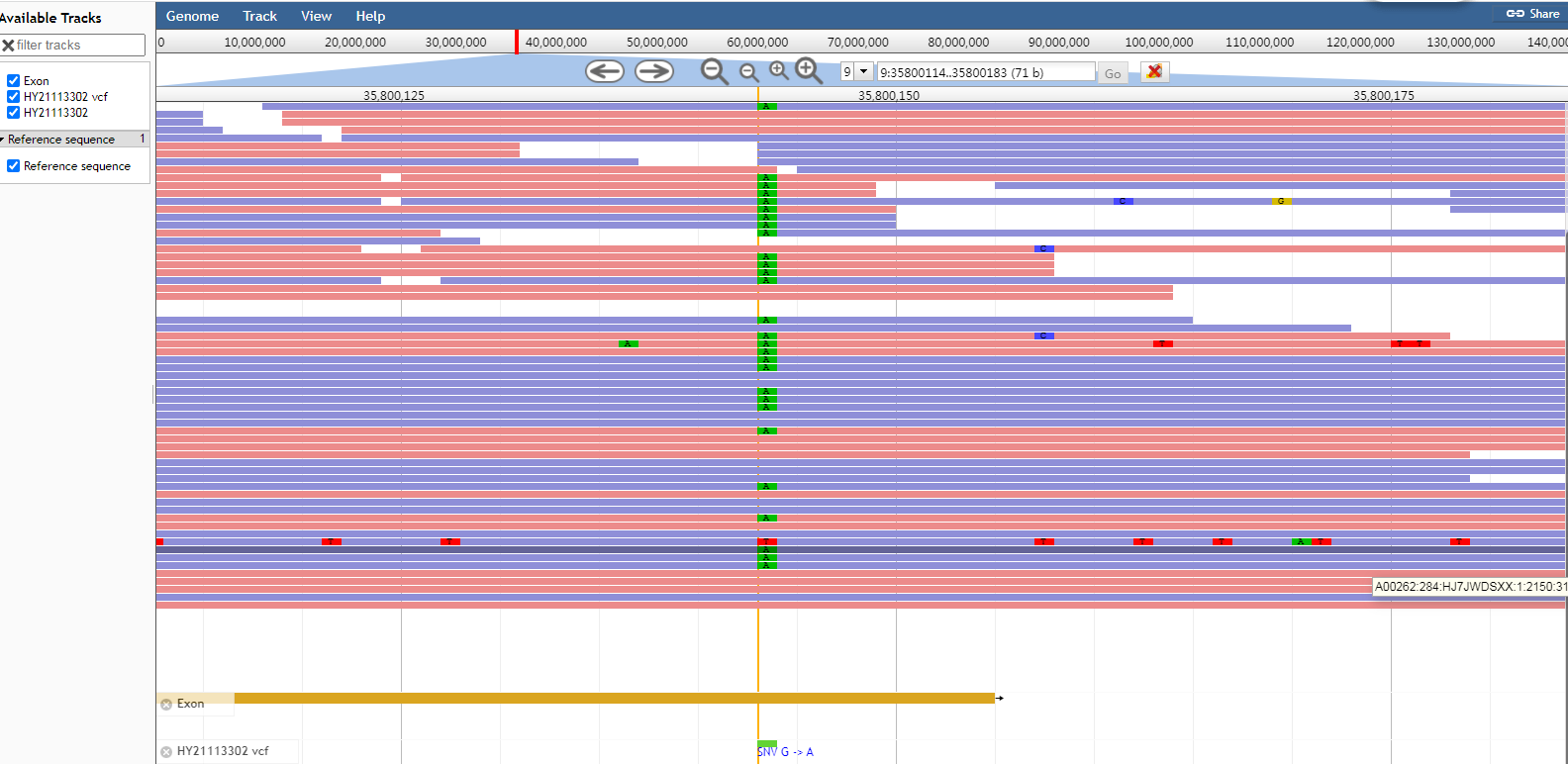


**Supplementary Figure 2. Screenshot of the exome alignment of c.2887+2T>C in the patient 1 (the black box).**

**
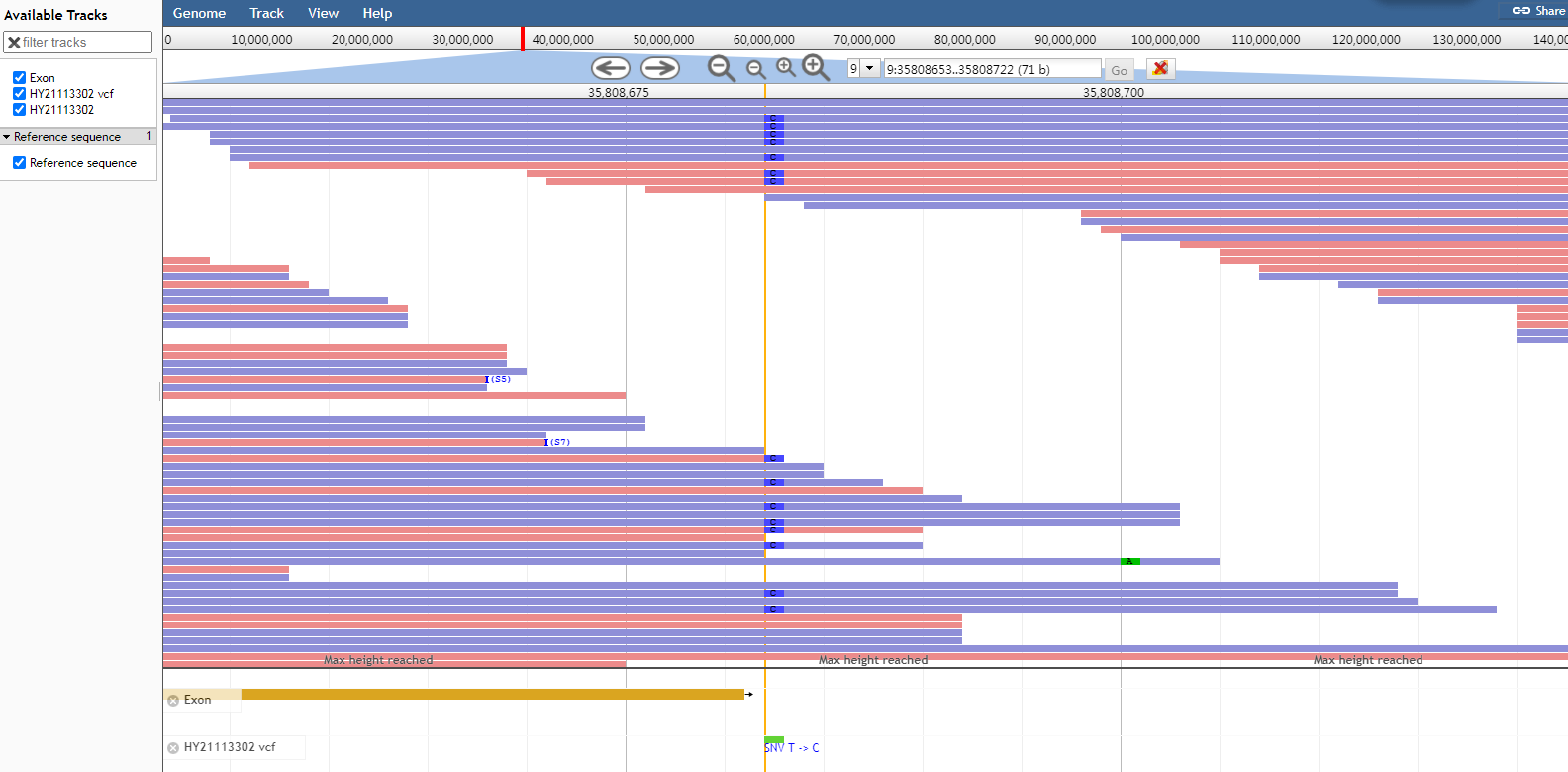
**

**Supplementary Figure 3. Screenshot of the exome alignment of c.329G>A p. (Arg110His) in the patient 2 (the black box).**


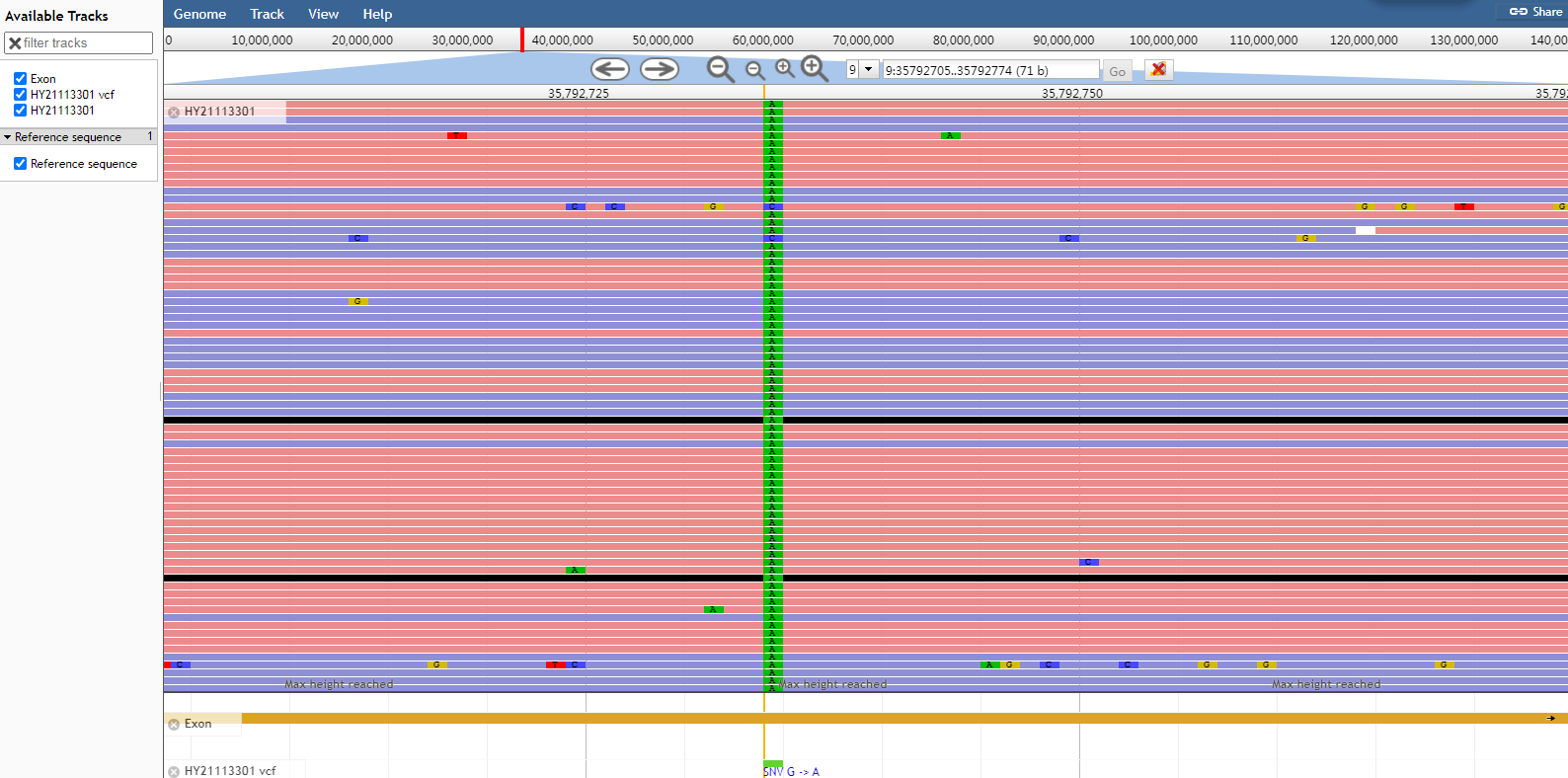


**Supplementary Figure 4. RT-PCR for c.2887+2T>C mutation of patient 1.**


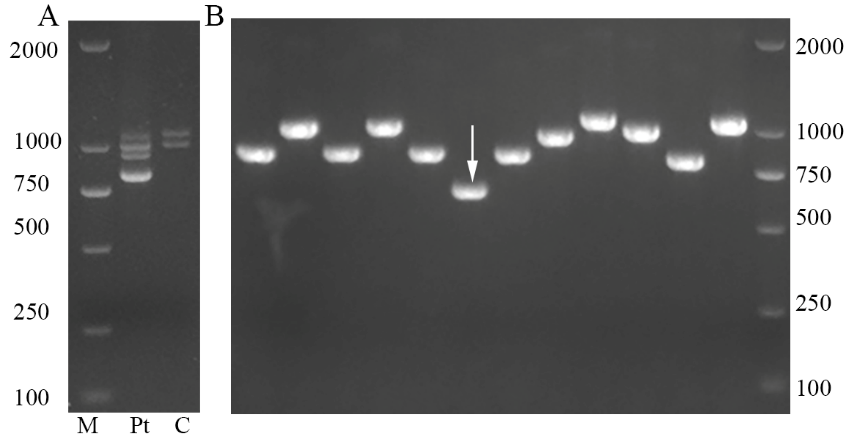


Supplementary Figure 4. RT-PCR for c.2887+2T>C mutation of patient 1. (A) RT-PCR analysis showing multiple forms of aberrant splicing PCR products (944bp, 826bp, 633bp) compared to normal sequence (1072bp, 1001bp). The band of 633bp is too weak to see. M: maker, Pt: Patient 1, C: Control. (B) Subcloning of the RT-PCR products in patient 1. The aberrant splicing form of 633bp is illustrated as white arrow.

**Supplementary Figure 5. RMSD of the ECD^WT^ dimer.**


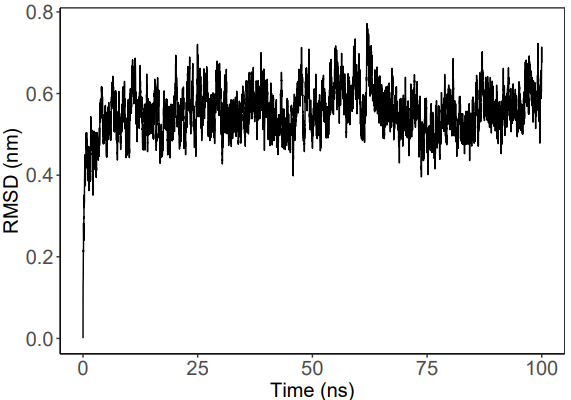


**Supplementary Figure 6. RMSD of the ECD^371Q^ dimer.**


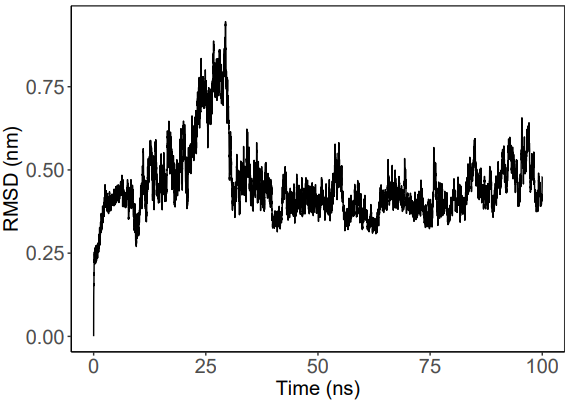


**Supplementary Figure 7. Distribution of all mutations contributed to AMDM.**


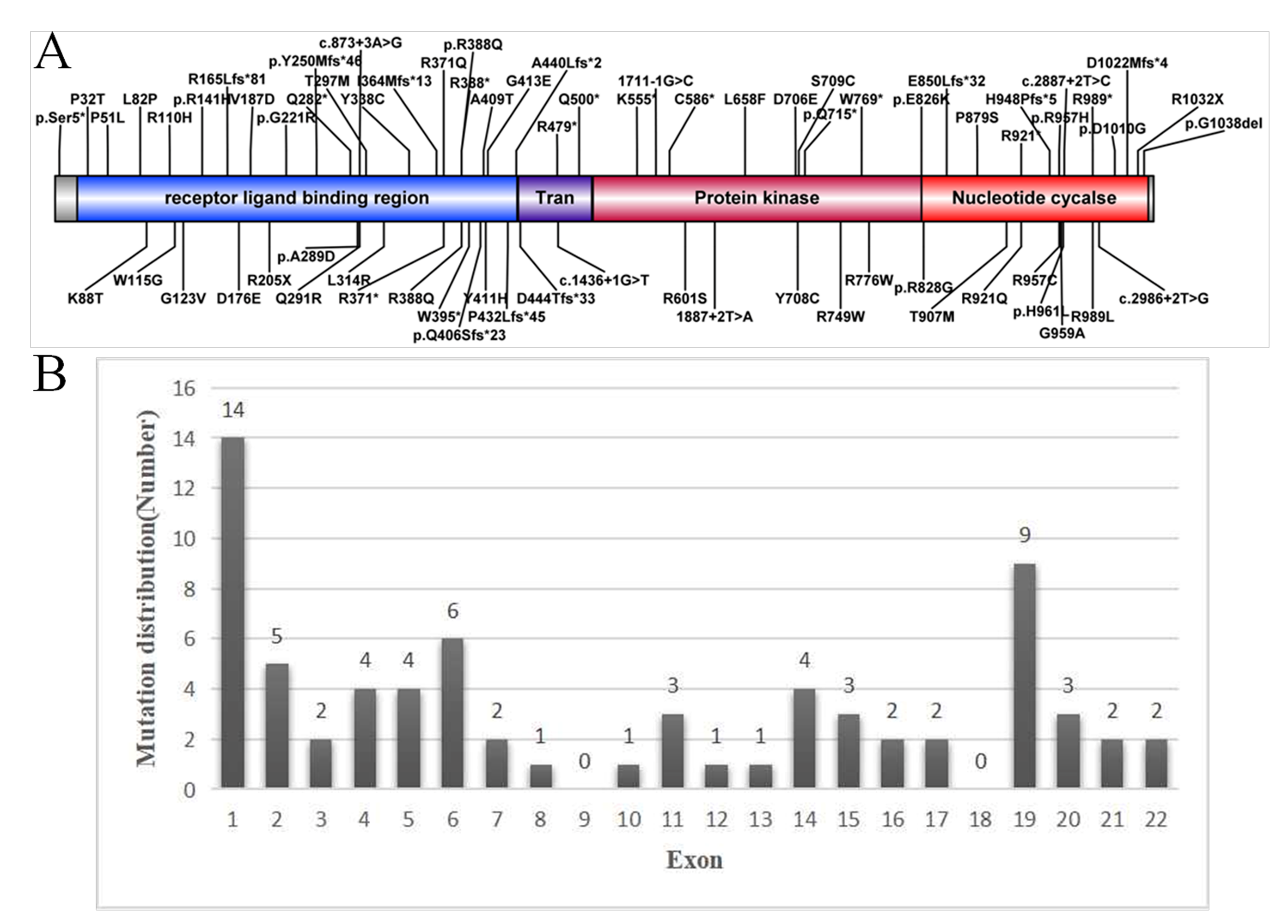


Supplementary Figure 7. Distribution of all mutations contributed to AMDM. (A) mutations were considered to be located in extracellular ligand-binding domain if they occurred between aa 23-441, transmembrane region if they occurred between aa 442-512, intracellular kinase homology domain if they occurred between aa 513-825, or carboxyl-terminal guanylyl cyclase domain if they occurred between aa 826-1042. (B) Distribution of mutations in exons of *NPR2.*
