# Supplementary material for: Novel Loss-of-Function Mutations in NPR2 Cause Acromesomelic Dysplasia, Maroteaux Type
Source: Front Genet. 2022 Mar 16;13:823861. doi: 10.3389/fgene.2022.823861 (PMC8967736; doi:10.3389/fgene.2022.823861)
Supplement: Supplementary file 1 [file DataSheet1.zip › 823861_SupMaterial/Data Sheet 1.ZIP!/raw datas/Figure 4B raw data.docx]

Subcloning of the RT-PCR product

Patient 1

Control
